# Paracrine effects of stem cells in wound healing and cancer progression

**DOI:** 10.3892/ijo.2014.2385

**Published:** 2014-04-11

**Authors:** JÜRGEN DITTMER, BENJAMIN LEYH

**Affiliations:** Clinic for Gynecology, University of Halle, Halle/Saale, Germany

**Keywords:** cancer stem cells, mesenchymal stem cells, angiogenesis, vascular endothelial growth factor, stromal cell-derived factor, microvesicles

## Abstract

Stem cells play an important role in tissue repair and cancer development. The capacity to self-renew and to differentiate to specialized cells allows tissue-specific stem cells to rebuild damaged tissue and cancer stem cells to initiate and promote cancer. Mesenchymal stem cells, attracted to wounds and cancer, facilitate wound healing and support cancer progression primarily by secreting bioactive factors. There is now growing evidence that, like mesenchymal stem cells, also tissue-specific and cancer stem cells manipulate their environment by paracrine actions. Soluble factors and microvesicles released by these stem cells have been shown to protect recipient cells from apoptosis and to stimulate neovascularization. These paracrine mechanisms may allow stem cells to orchestrate wound healing and cancer progression. Hence, understanding these stem cell-driven paracrine effects may help to improve tissue regeneration and cancer treatment.

## Contents

1. Introduction
2. Paracrine effects of stem cells in tissue regeneration
3. Paracrine effects of stem cells in cancer
4. Conclusions

## 1. Introduction

Stem cells are characterized by their ability to self-renew and their capacity to differentiate to specialized cell types (1). Stem cells are found in most tissues of the human body and are required to maintain tissue homeostasis (2). They are also engaged in wound healing (3). A recent work by Fuchs *et al* on hair follicle stem cells suggests that the more adult stem cells are present in the injured area the faster the wound is healing (4). This might be explained by an accelerated recruitment of differentiated cells as generated by a higher number of stem cells. However, there is evidence that besides differentiation capacity also paracrine functions of stem cells are important in wound healing (5).

A stem cell type that, for quite some time, is known to apply paracrine effects to orchestrate wound healing is the mesenchymal stem cell (MSC), a multipotent stromal progenitor cell residing preferentially in bone marrow and adipose tissue (6,7). MSCs are defined by their ability to differentiate to osteoblasts, chondroblasts and adipocytes, by plastic adherence and by a particular expression pattern of certain surface proteins (8,9). Strongly attracted to wounds, MSCs are mobilized by injuries which they enter to modulate inflammatory responses and stimulate tissue regeneration (10). MSCs are a heterogeneous population and can also emerge from pericytes or endothelial cells (11), which may help to accelerate local MSC recruitment. MSCs were originally reported to contribute to tissue repair by trans-differentiating into cells, such as epithelial cells or neurons, that are required to restore the injured tissue (12–15). However, later it became evident that their paracrine activities are more important for wound healing than their differentiation potential (11,16,17).

It is now well accepted that, also in cancer, stem-like cells, so-called cancer stem cells (CSCs), exist (18–21). These cells are thought to be responsible for tumor initiation and metastasis. As wounds that never heal (22) cancers resemble wounds in a number of aspects, e.g., in their ability to attract MSCs (23). CSCs are thought to contribute to tumor heterogeneity by generating different kind of differentiated cells. In breast cancer, CSCs can give rise to the so-called basal and luminal type of breast cancer cells (24). As suggested for adult stem cells, CSCs may have other functions besides recruitment of differentiated cells und may use paracrine activities to influence (tumor) tissue growth and maintenance. In this review, we will summarize the current knowledge on the importance of normal and cancer stem cells as producer of paracrine factors. Since there are a number of excellent reviews that address the paracrine functions of MSCs in wound healing and cancer (11,25–30), we focussed here on the paracrine effects of non-MSC stem cells and describe MSC paracrine activities only for comparative reasons.

There are many ways by which cells can communicate in a paracrine manner. One way is by proteins, such as growth factors or cytokines. MSCs secret a plethora of such proteins (28,29,31) some of which act as survival factors on neighboring (differentiated) cells, others stimulate angiogenesis. The cocktail of proteins that is secreted by cells is called the secretome (32). Besides the secretome, additional non-protein factors, such as lipids and RNAs, can be released from cells into the extracellular space. Some of these factors, in particular RNAs, may not leave the cell as soluble substances, but rather as cargos of microvesicles that are generated by the secreting cell. Microvesicles are circular fragments which can either be generated from endosomes (called exosomes; size range, 40–120 nm) or from the plasma membrane (called shedding vesicles; size range, 100–1,000 nm) (33–35). They can be distinguished from apoptotic bodies by their lack of DNA and histones. Both exosomes and shedding vesicles contain proteins of the lipid raft and lipids, such as cholesterol, as well as numerous soluble proteins and RNAs (mRNA and microRNA), e.g., in MSC-derived microvesicles, more than 700 proteins and ∼150 miRNAs have been identified (36,37). By interacting with microvesicles, cells can take up the microvesicular contents (37,38) and use them for biological activities. Microvesicular RNA may be of particular importance. RNA from microvesicles can be translated into proteins (39) and RNase treatment often abrogates the effect of microvesicles on recipient cells (40,41). Many effects of microvesicles have been described. Among them are inhibition of apoptosis, stimulation of stem cell activity or modulation of inflammatory responses (41–43).

## 2. Paracrine effects of stem cells in tissue regeneration

### Myocardial infarction

Cardiac stem cells have been shown to improve recovery of the myocard from ischemia. This has been linked to their ability to differentiate to cardiomyocytes to replace the damaged cells. However, a recent report demonstrated that the differentiation potential of these cells alone was not sufficient for this repair (44). The cardioprotective effect of the cardiac stem cells also strictly depended upon the activation of signal transducer and activator of transcription 3 (STAT3) in the myocard. STAT3 can be activated by stromal cell derived factor-1 (SDF-1), a chemokine secreted by cardiac stem cells and known to support regeneration of the myocardial tissue (45). Inhibition of SDF-1 secretion blocked recovery. SDF-1 has a dual function in myocard repair. It recruits stem cells to the infarcted heart (45) and improves the survival of cardiomyocytes (46) by decreasing caspase 3-dependent apoptosis (44). In the infarcted dog heart, recruitment of cardiac stem cells could be induced by administration of insulin growth factor-1 (IGF-1) and hepatocyte growth factor (HGF), two growth factors that stimulate the expansion of cardiac stem cells (47).

Besides cardiac stem cells, mesenchymal stem cells (MSCs) are able to improve post-ischemic recovery of the myocard (48). It was originally thought that multipotent MSC differentiate into cardiomyocyte-like cells to exert this effect, until it was found that the cocktail of proteins as secreted by MSC was sufficient for MSC-dependent recovery (5,49,50). Interestingly, like cardiac stem cells, MSCs induce STAT3 phosphorylation in the myocard (51). Moreover, toll-like receptor 4 (TLR 4)-deficient MSCs that induce much higher STAT3 activation were more effective in repairing the myocardial tissue than their wild-type counterpart. In the presence of MSC-conditioned medium (CM), also SDF-1 levels were higher in the infarcted heart (52). The SDF-1 level could be increased when the CM was taken from MSCs that had been forced to express vascular endothelial growth factor (VEGF). Part of the SDF-1 protein derived from the MSCs, part from the myocard. Hence, MSCs and cardiac stem cells may exert their cardioprotective effect via the same route and by using the same secretory protein(s). In a porcine model, it could be confirmed that MSCs, in this case generated from human embryonic stem cells, can improve recovery of the myocard from ischemia via factors they secrete (53). However, in this study, the cardioprotective effect was accompanied by decreased phosphorylation of Smad2, an effector of the transforming growth factor β pathway, and by reduced expression of caspase 3. In addition, the component responsible for this effect of the MSC-derived CM was found to be rather large, a complex of >1,000 kD. Later, a 20S proteasome, that copurifies with MSC-shedded exosomes, was identified as the likely candidate mediating MSC-dependent cardioprotection (54). The uptake of this proteosome by cardiomyocytes decreased the accumulation of misfolded proteins and may have therefore increased the survival of these cells. This is in agreement with the observation that MSC-derived CM upregulated anti-apoptotic protein Bcl2 in cardiomyocytes and protected them from hypoxia-induced apoptosis (55).

Additionally, MSCs may stimulate angiogenesis in the infarcted myocard. MSC-derived CM was shown to activate endothelial cells and to increase capillary density in the infarcted heart (50,56). Among the pro-angiogenic factors found in the secretome of MSCs are VEGF and basic fibroblast growth factor (bFGF) (50,55,57,58). Blocking VEGF and bFGF by antibody treatment could partly diminish recovery by MSCs (58). In addition to VEGF and bFGF, cysteine-rich angiogenic inducer 61 (Cyr61) has been identified as an important MSC-derived soluble factor that stimulates angiogenesis in the infarcted myocard (59). The anti-fibrotic activity of MSCs is also considered to contribute to the beneficial effect of these cells on the infarcted myocard. MSC-derived CM reduced cardiac fibrosis by inhibiting the proliferation of cardiac fibroblast and, thereby, decreasing the deposition of collagen I, II and III (60,61).

There are at least two more stem/progenitor cell types, the bone marrow-derived endothelial progenitor cell (EPC) and the skeletal muscle-derived stem cell (MDSC), which were shown to be capable of cardioprotection (62,63). When EPCs were transplanted into the myocard, again, myocardial expression of SDF-1 was found to be increased (62). In addition, EPS may stimulate angiogenesis in the myocard by secreting thymosin β4, a protein known to improve endothelial function (64). MDSCs were barely able to differentiate to cardiomyocytes, when implanted into the infarcted heart (65). Again it was their secretory activity that improved recovery from infarction. The major component of their secretome responsible for this effect was determined to be VEGF which stimulated angiogenesis. Blocking VEGF resulted in reduced neovascularization and adverse remodeling. Interestingly, mechanical stretching of MDSCs increased VEGF secretion. This finding, combined with the observation that mice that exercised after infarction showed higher myocardial VEGF levels and angiogenesis (66), may suggest that physical therapy after myocardial infarction improves recovery by increasing stem cell/VEGF-depending neovascularization (67).

In a recent study, the cardioprotective activities of cardiac stem cells, MSCs and EPCs were compared. Most effective in inducing myocyte differentiation and tube formation were cardiosphere-derived cells, a population of cells that contained cardiac stem cells and supporting cells (68). Compared to MSCs or bone marrow-derived mononuclear cells, these cells produced much higher levels of SDF-1, HGF and of the proangiogenic proteins VEGF and bFGF.

Duran *et al* asked the question if transplantation of cardiac and mesenchymal stem cells into the infarcted heart would change the cocktail of secreted factors (69). They first showed that, when cultured *in vitro*, both stem cell types secreted all of the 8 factors they had tested, including SDF-1 and VEGF. However, once transplanted into the infarcted heart, only VEGF and bFGF remained as the prominent proteins produced by both stem cell types. Along with the secretion of these two pro-angiogenic factors, both cell types stimulated neovascularization in the infarcted area which could not be attributed to differentiation of these stem cells to blood vessel cells. Surprisingly, SDF-1 was not found at any time point post-transplantation.

In summary, factors secreted by cardioprotective stem cells seem to have two major functions, i) to improve survival of cardiomyocytes; and ii) to stimulate neovascularization (Table I).

### Damage of the nervous system

Similar to the ischemic myocard, the ischemic brain requires stem cell-secreted factors for recovery. Here, again, the pro-angiogenic growth factor VEGF secreted by transplanted human central nervous system stem cells was found to be critical for stem cell-dependent repair of stroke-induced lesions (70). Neural stem cells also stimulated axonal transport and induced increased dendritic branching and length (71). The effect of neural stem cell on dendritic plasticity was at least partially dependent upon thrombospondins 1 and 2, two proteins secreted by the stem cells. This is in line with the observation that knockout of thrombospondin 1 and 2 in mice reduced functional recovery after stroke (72). Neural stem cells were also reported to improve repair of spinal cord injuries in rats. Implanted into the lesion area these cells enhanced axonal outgrowth (73). Neutrotrophic factors, such as nerve growth factors (NGF) and brain-derived neurotrophic factor (BDNF), were found to be secreted by the neural stem cells and were made responsible for this effect. Spinal cord injured rats also benefitted from CM generated by bone-marrow derived MSCs (74). Improved motor recovery in the presence of this medium was the consequence of less extensive lesions. Though MSCs secrete NGF and BDNF, protect neurons from apoptosis (74) and stimulate neurite outgrowth *in vitro* (75), MSC-CM seem to have no effect on axonal outgrowth *in vivo* (74). Rather, MSC-CM appears to exert its neuroprotective effect *in vivo* by stimulating angiogenesis. This was again at least partly dependent on VEGF.

### Kidney injury

Paracrine effects of stem cells also play a role in recovery from kidney injury. Tubular adult renal stem/progenitor cells (tARPC) have been reported to stimulate proliferation and to inhibit apoptosis of cisplatin-injured proximal tubular epithelial cells (76). This effect depended upon the secretion of inhibin A, an inhibitor of the TGFβ superfamily ligand activin known to inhibit renal tubulogenesis (77). Evidence was provided that inhibin A was transported to the tubular epithelial cells as RNA via microvesicles (76). Interestingly, inhibin A was only found in microvesicles shedded by tARPC that had encountered damaged tubular epithelial cells. For the recognition of apoptotic epithelial cells, toll receptor 2 (TLR2) was required. Also microvesicles shedded from bone marrow-derived mesenchymal stem cells were found to increase survival and proliferation of tubular cells after damage (40,41). As RNase treatment abrogated this effect, again the transfer of certain RNAs by the epithelial cells was made responsible for this process. Furthermore, the presence of CD44 and CD29 on the surface of these microvesicles were found to be crucial for the communication between the MSC-derived microvesicles and tubular cells. In addition, soluble factors, namely VEGF, IGF-1 and HGF, as secreted by MSCs may contribute to the renoprotective effect of MSCs. These factors may be responsible for the increased survival of endothelial cells as observed in the presence of MSCs (78,79). Interestingly, MSCs were found to attach to endothelial cells to form tubes in a cooperative manner (78).

In chronic kidney disease, exosomes from MSCs brought no improvement (80). However, non-fractionated CM from MSCs reduced disease progression and rescued renal function.

### Other injuries

Stem cell-secreted factors have also been shown to improve recovery of liver from cirrhosis (81). In this case, Wistar rats poisoned with dimethylnitrosamine were treated with or without CM from CD34^+^ haematopoietic stem cells. The CM from these cells injected into the tail vein significantly increased liver repair and animal survival by blocking caspase 3-dependent apoptosis of liver cells. Among the 32 factors identified in the CM of the CD34^+^ stem cells were a number of cytokines, including members of the CXCL chemokine family, known to be involved in wound healing. Liver regeneration is closely linked to CXC receptor 2 (82) which recognizes CXCL chemokines.

## 3. Paracrine effects of stem cells in cancer

### Glioma

CD133^+^ glioma cancer-initiating/stem-like cells are able to suppress immune responses against the tumor by inhibiting T-cell effector activity and stimulating that of T-cell suppressor cells (Tregs) (83). These activities depended on the presence of phosphorylated STAT3 in the cancer stem cells and on the ability of these cells to activate STAT3 in the immune cells. Since CM from the glioma CSCs was as effective as the CSCs themselves in inducing immunosuppression (84), it is likely that CSC-secreted factors are responsible for STAT3 activation. Among the factors present in the CSC-derived CM were transforming growth factor β1 (TGFβ1) and prostaglandin E2, two major secretory factors responsible for the immunosuppressive effects of MSCs (30). In addition, galectin-3, a β-galactoside-binding protein that in its soluble form can induce T-cell apoptosis (85), was found to be secreted by the glioma CSCs (84). Interestingly, galectin-3 is expressed by glioma cells, but not by astrocytes or oligodendrocytes (86). On its surface, the glioma CSCs also present the protein B7-H1 (84). This inhibitory co-stimulatory molecule inhibited T-cell proliferation through cell-cell interaction. These data indicate that glioma CSCs strongly contribute to the immunosuppression in gliomablastoma multiforme by paracrine effects as well as by mechanisms involving direct contacts with immune cells.

In addition to their immunosuppressive effect, glioma CSCs were found to stimulate angiogenesis. As a pro-angiogenic factor, CD133^+^ glioma CSCs secret substantial amounts of VEGF which leads to enhanced endothelial migration and tube formation (87). The level of secreted VEGF could be greatly enhanced by hypoxia. Forced overexpression of VEGF in CSCs also resulted in increased angiogenesis and tumor formation *in vivo* (88) confirming that CSCs can be a VEGF source to promote angiogenesis in glioma. Similar data were reported by Folkins *et al*, who compared glioma CSC high and low fractions (89). Besides VEGF, the CSC-high fraction also secreted SDF-1. Both VEGF and SDF-1 were necessary for the stimulatory effect of the CSC-high fraction on angiogenesis. Inhibition of either the VEGF receptor VEGFR2 or the SDF-1 receptor CXCR4 in endothelial cells equally blocked angiogenesis by CM from CSCs. CXCR4 is also highly expressed in glioma CSCs, where it stimulates VEGF secretion via the phosphoinositide 3-kinase (PI3K)/AKT pathway upon binding to SDF-1 (90). This suggests that SDF-1 has two functions in glioma CSC-driven angiogenesis: i) together with VEGF, it activates endothelial cells; and ii) it recruits more VEGF by stimulating its expression in glioma CSCs.

Interestingly, MSCs, which have been shown to stimulate angiogenesis in prostate cancer (91), suppress angiogenesis in glioma and hence inhibit glioma growth *in vivo* (92). Concomitantly, the expression of pro-angiogenic factors, such as bFGF, platelet-derived growth factor-BB (PDGF-BB) and IGF-1, were reduced suggesting that MSCs inhibited the secretion of these factors by the glioma cells. However, another study using glioma stromal mesenchymal stem-like cells (GS-MSLCs), which are MSC-like cells residing in glioma, demonstrated that MSCs are also able to promote angiogenesis (93). Apparently, the source the MSCs are isolated from is an important factor that determines the effect of MSCs in glioma (94).

### Renal cancer

Pro-angiogenic activities can also be attributed to CSCs isolated from renal cancer (95). These CD105-expressing CSCs stimulated angiogenesis by secreting exosome-sized microvesicles (96). CD105-positive, but not CD105-negative microvesicles, contained RNAs encoding angiogenic factors, such as VEGF. The CSC-derived microvesicles induced invasion of human vascular endothelial cells, protected them from apoptosis and promoted endothelial/tumor cell adhesion. They also stimulated angiogenesis in Matrigel plug assays *in vivo*. Treatment of lung endothelial cells with these microvesicles increased their expression of VEGF receptor and of matrix metalloproteinases 2 and 9. There is also evidence provided that these CSC-secreted microvesicles promote metastasis formation of renal cancer cells in the lung.

### Colon cancer

Also colon CD133^+^ CSCs support tumor survival by paracrine actions. The sensitivity of CD133^−^ non-CSC colon cancer cells to 5-fluorouracil and oxaliplatin was shown to strongly increase when interleukin-4 (IL-4), a cytokine present in colon cancer and absent in normal colon, was blocked by an IL-4 specific antibody (97). Interestingly, CD133^+^ CSCs were identified as the source of IL-4, although, in colorectal cancer, Th_2_ lymphocytes, the major producer of this inflammatory cytokine, are significantly increased in numbers (98). The CSCs themselves also benefitted from this cytokine as it participated in sustaining their chemotherapy resistance. Hence, CSC-derived IL-4 acted as both a paracine and autocrine survival factor in colon cancer. Blockage of IL-4 resulted in downregulation of anti-apoptotic proteins, such as Bcl-xL, suggesting that IL-4 protects colon cancer cells from cytotoxic drugs by inhibiting apoptosis. IL-4 has also been demonstrated to protect other cancer cell types, such as breast, bladder, prostate and thyroid cancer cells, from apoptosis (99,100). This suggests that IL-4 may be of general importance for cancers to gain therapy resistance. Emmink *et al* identified another way by which colon CSCs may contribute to therapy resistance of colon cancer (101). Comparing the secretome of colon CSCs with that of more differentiated colon cancer cells in the bulk tumor they found that CSCs secreted much higher levels of aldehyde dehydrogenase family 1, member A1 (ALDH1A1) and bleomycin hydrolase (BLMH), two enzymes able to detoxify chemotherapeutics. They could show that CSC-secreted ALDH1A1 and BLMH protected the colon cancer cells from cyclophosphamide and bleomycin, respectively.

### Ovarian cancer

Recently, ovarian CSCs have been reported to release CCL5 into the culture medium (102), a chemokine known to play a role in breast cancer metastasis and whose secretion can be triggered by co-culturing breast cancer cells with mesenchymal stem cells (103). CCL5 increased the migratory and metastatic potential of ovarian CSCs in an autocrine manner, but had little effect on non-CSC ovarian cancer cells. However, since the autocrine CCL5 feedback loop fueled expression of MMP-9 by CSCs, it is possible that secreted MMP-9, a protease involved in ECM degradation, facilitates invasion also of neighboring non-CSC tumor cells.

### Breast cancer

In breast cancer, the vast majority of studies on paracrine effects of stem cells have been done with MSCs which by heavily communicating with breast cancer cells via many soluble factors are able to promote tumor progression (26,104). Interestingly, MSCs may also affect breast CSCs. Liu *et al* demonstrated that IL-6-stimulated MSCs produce the chemokine CXCL7 which further fuels IL-6 secretion by breast cancer cancer cells (105). In the end, this feedback loop leads to the release of factors, such as IL-8, that cause the CSC pool to expand. In a different way, adipose-derived stem cells were found to increase the breast CSC population. By secreting PDGF-D, these stem cells induce epithelial-to-mesenchymal transition of breast cancer cells and, as a consequence, generate additional stem-like cancer cells (106).

In addition, breast CSCs may themselves be a provider of bioactive soluble factors. Comparative transcriptome analyses by serial analysis of gene expression, cDNA microarray and next generation sequencing of CD44^+^/CD24^−^ breast CSCs and bulk tumor cells revealed a highly active TGFβ pathway in CSCs compared to non-CSC breast cancer cells (107,108). Along with the activation of the TGFβ pathway, typical TGFβ target genes, such as plasminogen activator inhibitor-1 (PAI-1), were found to be highly upregulated in CSCs. PAI-1, a well-established unfavorable prognostic factor in breast cancer (109), is a secretory protein able to promote cellular migration and angiogenesis (110,111). Since PAI-1 secreted by MSCs is able to enhance migratory activities of cancer cells (112) (Dittmer *et al* unpublished data), it is reasonable to assume that CSC-secreted PAI-1 may also affect cell motility.

### Other cancers

Connections between CSCs and endothelial cells have been demonstrated for squamous tumor of the skin. CSCs in skin papillomas produce large amounts of VEGF not only to trigger angiogenesis by stimulating neighboring VEGFR2-expressing endothelial cells, but also to maintain their stemness in an autocrine manner via the VEGF co-receptor neuropilin 1 (113). Blocking the function of either neuropilin 1 in CSCs or of VEGFR2 in endothelial cells reduced both microvessel density and CSC population. Hence, cutaneous CSCs are propagated in perivascular niches, which are maintained by the VEGF produced by the stem cells themselves. Also CD133^+^ melanoma stem cells have been shown to produce pro-angiogenic factors, such as VEGF (114). In pancreatic cancer, CD133^+^ cancer stem cells have been found to be the major source of VEGF-C (115).

## 4. Conclusions

**Figure 1.**
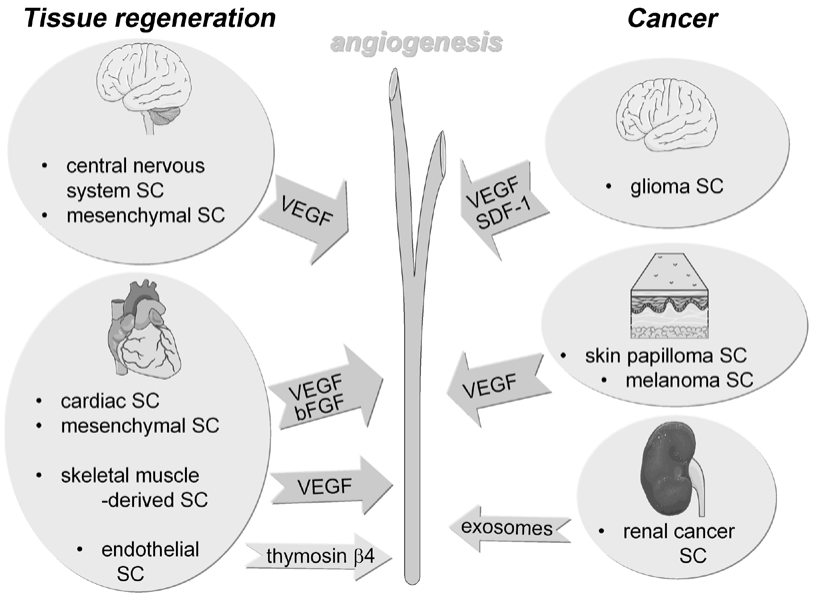
Pro-angiogenic effects of stem cells in tissue repair and cancer.

Though the importance of paracrine effects for the functions of mesenchymal stem cells in tissue repair and cancer is well established, we just start to appreciate paracrine activities of other adult stem cells and cancer stem cells. In the past, tissue-specific adult stem cells and cancer stem cells were only viewed as providers of new (differentiated) cells either to fill the gap that has been caused by cell loss or to fuel tumor growth, respectively. Now, a new theme is emerging which ascribe to these stem cells an additional regulatory function in tissue maintenance or tumor progression. The so-called damage-associated molecular pattern (DAMP) after acute kidney injury may be a good example that shows how much stem cell-derived factors are involved in tissue repair (116). It seems that adult stem cells orchestrate wound healing by releasing specific factors that inhibit apoptosis of damaged cells and stimulate angiogenesis. The secretion of pro-angiogenic factors by stem cells may be of particular importance, since this activity is shared by many adult stem cells and cancer stem cells and often found to be essential for the stem cell-driven tissue regeneration and stem cell-dependent tumor progression, respectively (Fig. 1). Since delivery of oxygen and nutrients is essential for cell survival timely angiogenesis in tissue repair and cancer progression is a critical event. Stem cells may coordinate tissue repair/cancer progression by generating new cells and, by stimulating angiogenesis, simultaneously supplying these cells with the substances needed for survival. It is intriguing that endothelial cells are often in close contact with stem cells. One example is the haematopoietic stem cells which are positioned next to endothelial cells when residing in the endothelial niche in the bone marrow and whose expansion is dependent on endothelial cells (30). Also glioma stem cells are residing in endothelial niches (117) which seem to be of mutual benefit for both cell types (118). Perivascular niches have also been found to regulate dormancy of breast cancer cells (119) and maintain CSC populations in skin cancer (113). In addition, endothelial cells may be strongly involved in cancer metastasis (120). The link between cancer stem cells and endothelial cells may theoretically open new evenues to treat cancer stem cells that are usually resistant to chemotherapeutics and whose population may even expand in the presence of these drugs (121). Anti-angiogenic drugs may dislodge cancer stem cells from their endothelial feeding layer and stop them from growing and differentiating. However, anti-angiogenic drugs, such as anti-VEGF, have been tested for some time in clinical trials to suppress blood supply to the tumor and showed limited success for several reasons, e.g., because hypoxia was induced that then fueled cancer progression (122). More knowledge is required to understand the role of the cancer stem/endothelial cell interaction in cancer progression to find specific drugs that interfere with this kind of cell-cell communication.

In general, knowing that paracrine effects of stem cells strongly contribute to tissue repair and cancer may help to find new ways of therapeutical interventions to facilitate tissue regeneration and to improve cancer treatment, respectively.

## Figures and Tables

**Table I.** Paracrine actions of stem cells in tissue regeneration and cancer.

Lesion	Stem cell (SC)/progenitor cell (PC) type	Secreted factor	Function	Comment	Refs.
Myocardial infarction	Cardiac SC	SDF-1	Survival of cardiomyocytes	Induces STAT3 activation	(44)
		VEGF, bFGF	Angiogenesis		(69)
	Mesenchymal SC	CM	Myocard repair	Induces STAT3 activation	(51)
		VEGF, bFGF, Cyr61	Angiogenesis		(58,59,69)
		20 S proteasomes via exosomes	Survival of cardiomyocytes	Reduces accumulation of misfolded proteins	(54)
	Endothelial PC	SDF-1	Myocard repair		(62)
		Thymosin β4	Improvement of endothelial function		(64)
	Skeletal muscle-derived SC	VEGF	Angiogenesis	Higher VEGF levels by mechanical stretching	(65,66)
Stroke	Central nervous system SC	VEGF	Neovascularization		(70)
	Neural PC	Thrombospondin 1 and 2	Higher axonal transport and dendritic branching		(71)
Spinal cord injury	Neural PC	NGF, BDNF	Stimulation of axonal outgrowth		(73)
	Mesenchymal SC	VEGF	Angiogenesis		(74)
Acute kidney injury	Tubular adult renal PC	Inhibin A, microvesicles	Survival and proliferation of tubular cells	Inhibin A probably transmitted via microvesicles	(76)
	Mesenchymal SC	Microvesicles	Survival and proliferation of tubular cells		(40)
Chronic kidney injury	Mesenchymal SC	CM	Reduction of tubular and glomular damage	Exosomes are not involved	(80)
Liver cirrhosis	Haemotopoietic SC	CM	Survival of liver cells	CXCL chemokines may be involved	(81)
Glioma	CD133^+^ glioma SC	CM	Immunosuppression	Requires STAT3 activation in CSCs	(83,84)
		VEGF, SDF-1	Angiogenesis		(88,89)
Colon cancer	CD133^+^ colon SC	IL-4, ALDH1A1, BLMH	Chemoresistance		(97,101)
	Mesenchymal SC	PAI-1	Stimulates migration		(112)
Skin papillomas	Skin papilloma SC	VEGF	Angiogenesis, maintains stemness		(113)
Renal cancer	CD105+renal cancer SC	Exosomes	Angiogenesis, lung metastasis	Exosomes contain VEGF-RNA	(96)

ALDH1A1, aldehyde dehydrogenase family 1, member A1; BDNF, brain-derived neurotrophic factor; bFGF, basic fibroblast growth factor; BLMH, bleomycin hydrolase; CM, conditioned medium; Cyr61, cysteine-rich angiogenic inducer 61; IL-4, interleukin-4; NGF, nerve growth factor; PAI-1, plasminogen activator inhibitor-1; SDF-1, stromal cell derived factor-1; STAT3, signal transducer and activator of transcription 3; VEGF, vascular endothelial growth factor.
